# Editorial: Factors Promoting Development of Fibrosis in Crohn’s Disease

**DOI:** 10.3389/fmed.2017.00160

**Published:** 2017-09-26

**Authors:** Jennifer Bailey

**Affiliations:** ^1^Bristol Veterinary School, University of Bristol, Bristol, United Kingdom

**Keywords:** Crohn’s disease, fibrosis, inflammatory bowel disease, strictures, extracellular matrix

**Editorial on the Research Topic**

**Factors Promoting Development of Fibrosis in Crohn’s Disease**

Crohn’s disease is a form of inflammatory bowel disease, which results in areas of chronic inflammation at any point in the gastrointestinal tract. Intestinal fibrosis is a common but debilitating complication of Crohn’s disease for which currently there is no medical treatment. In a number of cases, significant fibrosis is already present at the time of disease diagnosis (1), and 40–70% of Crohn’s disease patients require surgery in the 10 years following diagnosis, with the development of fibrotic strictures being the leading indication for surgery in small bowel disease (2). Fibrosis occurs due to excessive deposition of extracellular matrix (ECM), particularly fibrous collagen, and dysregulated turnover of ECM. Affected tissue becomes hardened and thickened, leading to a loss of function. Fibrogenesis in Crohn’s disease is a multifactorial process, influenced by immunological, environmental, genetic, and disease-related factors (3–6). It is not currently known how or why fibrosis develops in such a high population of patients. Fibrosis follows the path of inflammation and has not been seen in areas unaffected by inflammation; however, inflammation is not required to propagate fibrosis once it has been initiated (7). This Research Topic brings together experts in the field to discuss recent advances in the pathogenesis of fibrosis and future perspectives for detection, treatment, and prevention of the condition.

Rogler and Hausmann introduce the topic with a review on the main factors that promote development of fibrosis in Crohn’s disease. They discuss new findings that challenge the current dogma in this area and highlight the importance of these when developing novel treatment strategies. It is clear that we need to expand our horizons when we consider which cells contribute to fibrosis and look beyond primary mesenchymal cells. While fibroblasts and smooth muscle cells are significant contributors to fibrosis, increasing evidence suggests a role for epithelial and endothelial cells *via* epithelial or endothelial-to-mesenchymal transition (8–10). Cytokines have long been known as key players in fibrogenesis, with those produced during the inflammatory response, and found at increased levels in the inflamed IBD gut contributing. In particular, transforming growth factor-β (TGF-β) and tumor necrosis factor-α (TNF-α) have been extensively studied and both stimulate collagen accumulation and mesenchymal cell activation (11). However, these same cytokines are also capable of stimulating both epithelial and endothelial-to-mesenchymal transition (8, 12) thus their pro-fibrotic effects extend further than originally thought.

The role of cytokines in fibrogenesis has been reviewed in depth in this Research Topic by Curciarello et al. Cytokines such as TGF-β, IL-1β, TNF-α, and IL-17A can have a direct effect on mesenchymal cells, preventing their migration away from the site of fibrogenesis, activating them to produce ECM components such as collagen, inducing their proliferation, and stimulating production of tissue inhibitors of matrix metalloproteinases, which prevent the normal breakdown of collagen (13–16). Cytokines such as IL-13, IL-6, and IL-33 contribute to fibrogenesis *via* an indirect effect on mesenchymal cells; for example, they prevent synthesis of MMPs and induce secretion of pro-fibrotic cytokines, such as TGF-β, by other cell types which are then capable of stimulating collagen deposition (17–20).

There are currently no medical therapies available to treat existing fibrosis. While major advances in surgical strategies have been made in recent years, such as strictureplasty, these procedures are not without risks and many patients prefer to avoid surgery. Early detection and intervention are key to minimizing damage but, as previously mentioned, fibrosis develops silently, demonstrated by the fact that a number of patients will already have significant fibrosis at the time of diagnosis (1). The ability to rapidly screen newly diagnosed patients to predict which already have fibrosis, or are at significant risk of developing fibrosis, could be key to improving outcomes for patients in the future. James and Tyrrell-Price discuss a recent publication on the accuracy of hybrid positron emission tomography (PET)/magnetic resonance-enterography (MR-E) and PET/computed tomography-enterography (CT-E) for the detection of fibrosis in Crohn’s disease (21). Differentiation between fibrotic strictures and inflammatory strictures is vital since treatment protocols differ between the two states; inappropriate classification could lead to unnecessary surgery or futile attempts at medical therapy. This study has evaluated these two radiological techniques to determine their effectiveness in predicting which patients will respond to medical therapy and which require surgical intervention. In this small study, PET/MR-E showed greater success in accurately detecting fibrosis than PET/CT-E; however, while these data are encouraging and indicate that PET/MR-E may be a useful diagnostic tool, current low availability and high cost prohibit its use as a routine screening procedure.

Genetic factors are likely to play in a role in the susceptibility of an individual to developing fibrotic disease. If these factors can be determined, it could unlock new treatment pathways or be used to predict the course of disease in newly diagnosed patients. Verstockt and Cleynen have reviewed the current knowledge of genetic influences on fibrogenesis in Crohn’s disease. Several genes have been implicated in the pathogenesis of Crohn’s disease; however, very few have been identified as independent risk factors for developing fibrostenotic disease. The most studied genetic predictor for fibrotic disease is nucleotide-binding oligomerization domain-containing protein 2 (*NOD2*). While a link has been found between *NOD2* and fibrostenotic disease in a number of studies (22–26), other groups have been unable to find this association (6, 27); therefore, the picture is unclear, and NOD2 genotyping in all patients is unjustified at present.

A major challenge for the future will be to develop novel medical therapies to treat and prevent fibrosis to reduce reliance on surgical techniques. Traditionally, fibrosis has been thought of as irreversible; however, this may not be the case; fibrosis has been shown to be reduced following strictureplasty (28), giving hope for new treatment strategies. Excellent progress has been made in the field of surgery but continuation of investigation into the processes that stimulate fibrogenesis and development of animal models with which to trial candidate therapies are critical if we are to achieve the holy grail that is medical treatment of fibrosis.

## Author Contributions

JB conceived and wrote the manuscript alone.

## Conflict of Interest Statement

The author declares that the research was conducted in the absence of any commercial or financial relationships that could be construed as a potential conflict of interest.
